# Published and not fully published double-blind, randomised, controlled trials with oral naratriptan in the treatment of migraine: a review based on the GSK Trial Register

**DOI:** 10.1007/s10194-011-0327-3

**Published:** 2011-03-25

**Authors:** Peer Carsten Tfelt-Hansen

**Affiliations:** Department of Neurology, Danish Headache Center, Glostrup Hospital, University of Copenhagen, Glostrup, Denmark

**Keywords:** Naratriptan, Unpublished clinical trials, Migraine

## Abstract

Naratriptan 2.5 mg is now an over-the-counter drug in Germany. This should increase the interest in drug. The GSK Trial Register was searched for published and unpublished double-blind, randomised, controlled trials (RCTs) concerning the use of naratriptan in migraine. Only 7 of 17 RCTs are published in full. Naratriptan 2.5 mg is superior to placebo for acute migraine treatment in 6 RCTs, but inferior to sumatriptan 100 mg and rizatriptan 10 mg in one RCT each. This dose of naratriptan has no more adverse events than placebo. Naratriptan 1 mg b.i.d. has some effect in the short-term prophylactic treatment of menstruation-associated migraine in 3 RCTs. In 2 RCTs, naratriptan 2.5 mg was equivalent to naproxen sodium 375 mg for migraine-related quality of life. Naratriptan 2.5 mg (34% preference) was superior to naproxen sodium 500 mg (25% preference). Naratriptan 2.5 mg is better than placebo in the acute treatment of migraine. The adverse effect profile of naratriptan 2.5 mg is similar to that of placebo. The efficacy of naratriptan 2.5 mg versus NSAIDs is not sufficiently investigated. Naratriptan, when available OTC is a reasonable second or third choice on the step care ladder in the acute treatment of migraine.

## Introduction

In Germany, the 5-hydroxytryptamine (5-HT)_1B/1D_ receptor agonist naratriptan is an over-the-counter (OTC) drug, most likely because of its excellent tolerability [1–3]. The time to maximum blood concentration is 2 h for oral naratriptan, and 1.5 h for oral sumatriptan [2, 4]. The oral bioavailability of naratriptan is 74%, and much higher than the 14% availability of sumatriptan [4]. The elimination half-lives of naratriptan and sumatriptan are 5.5 and 2 h, respectively [4]. Naratriptan 2.5 mg tablets have a placebo-like tolerability profile and are associated with a low incidence of headache recurrence [5]. Thus, the 2.5-mg dose offers some advantages over other 5-HT_1B/1D_ agonists, and naratriptan has been called the “gentle triptan” [5].

In 1998, the Ethics Subcommittee of the International Headache Society [6] stated that the “responsibility for publication cannot be separated from the ethical responsibility of the investigator”. The Subcommittee agreed that “scientists have an ethical obligation to submit creditable research results for publication, and should not enter into agreements that interfere with their control over the decision to publish.” As a general rule, every methodologically sound, randomised, controlled trial should be published to allow an evaluation of the results; publication solely as an abstract or in non-peer-reviewed supplements is unacceptable [6].

Recently, I reported on six unpublished randomised controlled trials (RCTs) with sumatriptan [7]. These RCTs were found in the GlaxoSmithKline (GSK) Trial Register, and I became aware of unpublished RCT with naratriptan. Because naratriptan is now becoming an OTC drug in some countries, a review of all RCTs is relevant. In the present review of 17 double-blind, randomised, controlled trials (RCTs) in the naratriptan part of the GSK Trial Register, it was remarkable that less than half of these RCTs were fully published in peer-reviewed journals.

## Methods

The GSK Register (http://www.gsk-clinicalstudyregister.com/) for naratriptan, which consists of 47 phase I to phase IV clinical studies, was searched for double-blind RCTs with oral naratriptan for migraine treatment regardless of the dose of naratriptan. The following data were extracted from the summary reports of each double-blind RCT (see Table 1): (1) doses of naratriptan, doses of other drugs, and the use of placebo; (2) number of centers; (3) total number of patients; (4) full publication (yes/no); (5) publication as an abstract (yes/no); and (6) reported results (either from the summary or from the full publication).

**Table 1:** Overview of 17 randomised, controlled trials with oral naratriptan for migraine treatment found in the GSK Trial Register (http://www.gsk-clinicalstudyregister.com/)

Drugs used [reference] (protocol code in GSK register)	Number of centers	Total number of patients (study design)	Full publication	Abstract(s)	Results
Na 2.5 mg vs. naproxen sodium (NS) 275 mg [8] [S2WA4003]	19	168 (P)	–	–	Na and NS had similar effect on MRQL
Na 2.5 mg vs. NS 275 mg [9] [S2WA4004]	20	171 (P)	–	–	Na and NS had similar effect on MRQL
Na 2.5 mg vs. NS 500 mg [10] [S2W40010]^a^	70	456 (C)	–	–	Preference: Na (34%) > NS (26%)^c^
Na 2.5 mg vs. PL in mild MAM [11] [S2W40031]	152	229 (P)	+	–	Na (58% headache relief) > PL (30% headache relief)
Na 1 mg b.i.d. vs. placebo for MAM [12] [S2W40012]	51	187 (P)	–	+	Without MAM: Na 38% > PL 29%
Na 1 mg b.i.d. vs. PL [13] [S2W40024]	61	236 (P)	–	+	Without MAM: Na 34% > PL 24%
Na 0.1, 0.25, 1.0, 2.5 mg vs. PL [14] [S2WA3001]	54	613 (P)	+	+	Na 1 mg (50%) and 2.5 mg (60%) > PL (34%) for headache relief at 4 h
Na 0.25, 1.0, and 2.5 mg vs. PL [15] [S2WA3003]	50	602 (C)	+	+	Na 1.0 mg (57%) and 2.5 mg (68%) > PL (33%) for headache relief after 4 h
Na 0.25, 1.0, and 2.5 mg vs. placebo in adolescent migraine [16] [S2WA3012]	44	300 (P)	–	+	Na 0.25 mg (72%), 1.0 mg (67%) and 2.5 mg (64%) were similar to PL (65%) for headache relief after 4 h
Na 2.5 mg vs. PL in pt not responding to sumatriptan [17] [S2WA4002]	57	206 (382) (P)^b^	+	+	Na (47%) > PL (20%) for headache relief after 4 h
Na 2.5 mg b.i.d. vs. PL in transformed migraine [18] [S2WA4005]	11	170 (P)	–	–	Na (13%) similar to PL (17%) for no headache on days 13 and 14.
Na 1 mg and 2.5 mg b.i.d. vs. PL in MAM [19] [S2WA4006]	18	206 (P)	+	+	Median MAM over 4 menstruation: PL = 4, 1 mg = 2, 2.5 mg = 3. Na 1.0 mg < PL (*p* = 0.011)
Na 2.5 mg vs. sumatriptan 100 mg in recurrence prone patients [20] [S2WB3011]	34	236 (C)	+	+	24 h overall efficacy: Na (40%) similar to sumatriptan (35%)
Na 0.1 mg, 0.25 mg, 1.0 mg, and 2.5 mg vs. sumatriptan 100 mg vs. placebo [21] [S2WB3002]	113	1,141 (942) (P)^c^	^−^	+	Na 1.0 mg (52%) and 2.5 mg (66%) > PL (27%) for headache relief at 4 h, sumatriptan (76%) was superior to all Na doses
Na 2.5 mg vs. sumatriptan 50 mg in patients who relapse from sumatriptan (100 mg orally or 6 mg subcutaneously) [22] [S2WB4001]	66	464 (C)	–	–	Satisfaction with overall effectiveness: very satisfied or satisfied: Na (52%) similar to sumatriptan (48%)
Na 5 mg and 10 mg vs. placebo [23] [S2WB2003]	10	90 (P)	–	–	Headache relief at 4 h: Na 5 mg (89%) and 10 mg (72%) > PL (33%)
Na 1, 2.5, 5, 7.5, and 10 mg vs. sumatriptan 100 mg and vs. PL [24] [S2WB2004]	74	637 (P)	+	+	For headache relief at 4 h all doses of Na (64%, 63%, 65%, 80%, and 80%) were superior to PL (39%). Na 7.5 mg and 10 mg were similar to sumatriptan 100 mg (80%), which was superior to Na 1, 2.5, and 5 mg

*C* crossover, *P* parallel group, *Na* naratriptan, *NS* naproxen sodium, *MRQL* migraine-related quality of life, *PL* placebo, *MAM* menstruation-associated migraine

^a^Patients dissatisfied with simple analgesics in the treatment of migraine attacks

^b^Number of patients treated with a single-blind dose of sumatriptan 50 mg

^c^Number of patients completing and treating 3 attacks

^d^Complete headache relief after 4 h was 39% in both treatment groups

## Results

All RCTs were multicenter RCTs (Table 1). Twelve out of 17 RCTs [8–24] were placebo-controlled, and in 5 RCTs [10, 20–22, 24] there was a presumably active comparator.

The median number of participating centers per RCT was 51 (range 10–152). In these 17 RCTs, the median number of patients was 236 (range 168–1,141). Seven RCTs [11, 14, 15, 17, 19, 20, 23] were fully published in a peer-reviewed journal. Ten RCTs [12–17, 19, 21, 23, 28] were published as an abstract, and 6 RCTs [8–10, 18, 22, 23] were never published.

Naratriptan 2.5–10 mg was superior to placebo for acute migraine treatment in all 6 RCTs in adults [11, 14, 15, 17, 21, 23], whereas naratriptan 2.5 mg was not superior to placebo in adolescents [16]. In 3 RCTs [11–13], short-term prophylaxis with naratriptan 1 mg b.i.d was superior to placebo for menstruation-associated migraine (MAM). In 2 RCTs [8, 9], naratriptan 2.5 mg was equivalent to naproxen sodium 375 mg for migraine-related quality of life. Naratriptan 2.5 mg (34% preference) was superior to naproxen sodium 500 mg (25% preference) [10]. Naratriptan 2.5 mg [66% headache relief (HR) at 4 h] was inferior to sumatriptan 100 mg (76% HR at 4 h) in one RCT [21], whereas the higher 10-mg dose of naratriptan (80% HR at 4 h) was quite similar to sumatriptan 100 mg (80% HR at 4 h) [24].

## Discussion

A recent review on reporting bias in clinical trials [25] concluded that “the prevalence of incomplete outcome reporting is high”. In addition, when all randomised controlled trials (RCTs) are not published, it results in publication bias, which can be considerable and can distort available evidence [26].

Publication of well-conducted RCTs is primarily the responsibility of the clinical investigators. However, all the RCTs on naratriptan were multicenter trials (range 10–152 centers) and the pharmaceutical company, GSK was most likely fully in control of both the conduct and the publication of the RCTs. Seven pivotal RCTs [11, 14, 15, 17, 19, 21, 23] were fully published, but one was not [21] (see Table 1). The reasons for not publishing the 10 other RCT in full remains unknown.

**Fig. 1:**
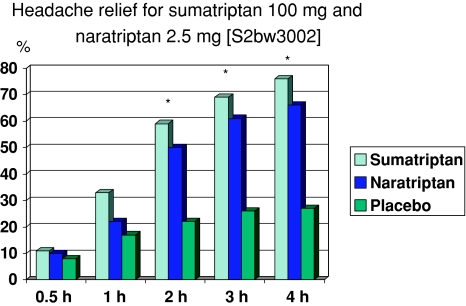
The mean headache relief for sumatriptan 100 mg, placebo, and naratriptan 2.5 mg up to 4 h in one not-fully published RCT [21]. *p < 0.05 for difference between naratriptan versus sumatriptan

**Fig. 2:**
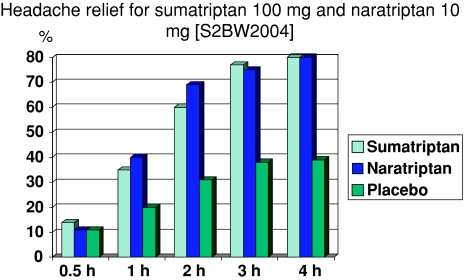
The mean headache relief for sumatriptan 100 mg, placebo, and naratriptan 10 mg up to 4 h in one RCT [23]

Notably, the 2,729 patients included in the fully published RCTs represent less than half (45%) of the 6,112 patients participating in the naratriptan trial program. When migraine patients were recruited to an RCT, which may include placebo or a less effective drug, for up to 4 h, it should be an obligation to publish the results in a peer-reviewed journal. The conduct of RCTs solely for registration purposes, without full publication, should be avoided. The data presented in this review should be and are now in the public domain. They confirm, as expected, that naratriptan is superior to placebo as was also found in meta-analyses [1–3]. It should be noted, however, that headache relief (a decrease in headache from moderate or severe to none or mild) was first measured after 4 h because naratriptan is generally held to be a slow-acting triptan [5]. The “slow onset of action” (a delay in onset) of naratriptan was apparently confirmed in one RCT (see Fig. 1) [21]. After 2 h, the headache relief for patients taking sumatriptan 100 mg and for naratriptan 2.5 mg were 59 and 50%, respectively. After 2 h, the curves for headache relief were parallel, with the endpoint headache relief at 4 h for sumatriptan 100 mg at 76% and naratriptan 2.5 mg at 66%. Thus, it is difficult to judge the speed of onset of action of the two drugs when the final endpoints are different [27]. In contrast, in an RCT using the higher 10-mg dose of naratriptan versus 100 mg sumatriptan, the endpoint of 4-h headache relief was similar, at 80%, for both groups [24]. As shown in Fig. 2, there is no difference in the “onset of action” when equipotent doses of the two drugs are used. Thus, naratriptan is not a slow-acting drug per se.

Naratriptan 1 mg b.i.d was superior to placebo for short-term prophylaxis of MAM in 3 RCTs, and naratriptan halved the MAM in one RCT [19], whereas in 2 RCTs 36% were without MAM versus 27% for placebo [12, 13]; these results suggest a modest effect of short-term prophylaxis with naratriptan in MAM.

Naratriptan was evaluated in 3 RCT versus the NSAID, naproxen sodium [8–10] (see Table 1). In two RCTs, naratriptan was not superior to naproxen sodium 375 mg for migraine-related quality of life [8, 9]. In one RCT, more patients (34%) preferred naratriptan than naproxen sodium 500 mg (25%) [10]. It is unclear why naratriptan 2.5 mg is similar to naproxen 375 mg but better than the higher 500-mg dose of naproxen sodium. It may be due to the design of the studies.

In conclusion, the investigators are obligated to the migraine patients participating in the trials, who often endured placebo administration for 4 h, to ensure that the results of well-conducted, randomised trials are published in peer-reviewed journals. This can be difficult when there are multicenter trials with many investigators, and I suggest that investigators choose a publication committee among themselves, e.g. one from each country or each region.

In this review, all double-blind RCTs with oral naratriptan in the treatment of migraine are presented [8–24]. This does not change the overall picture of an effective and well-tolerated triptan [3]. Whereas naratriptan is superior to placebo, the 2.5-mg dose chosen is less effective than sumatriptan 100 mg (2 and 4 h) (see Table 1), and rizatriptan 10 mg (2 h) [28]. The inferiority of naratriptan versus most other triptans at 2 h has been shown in meta-analyses [1, 2, 4], and this has been ascribed to a slow action of naratriptan [5]. The apparent slow onset of naratriptan 2.5 mg is, however, a matter of dose (see above).

When naratriptan 2.5 mg became an OTC drug, the question of its efficacy compared with other OTC drugs and NSAIDs became relevant. Naratriptan was not compared with paracetamol or aspirin, which in an effervescent form (52% headache relief) was found to have similar efficacy as sumatriptan 50 mg (46% headache relief) in a meta-analysis [28]. There are 2 RCTs comparing naratriptan 2.5 mg with naproxen sodium 275 mg (Table 1). Unfortunately, no placebo-control was used in the RCTs, but no differences between the active drugs were observed. Patients’ preference for naratriptan (34%) was marginally superior to naproxen sodium (26%) (Table 1) in patients dissatisfied with simple analgesics [10]. What is missing is a comparative RCT of naratriptan 2.5 mg (50% headache relief Table 1), and lysine acetylsalicylate and metoclopramide, which showed headache relief of 56% in 2 RCTs [29, 30]. Until such comparative RCTs become available, one cannot, based on the available evidence, recommend what migraine patients should try next when they are dissatisfied with simple analgesics. Naratriptan is a reasonable OTC choice as second or third choice on the step care ladder.
